# Clinicopathologic correlates of Ewing (FET::ETS-rearranged) sarcoma and non-Ewing undifferentiated small round cell sarcomas across skeletal and extraskeletal sites

**DOI:** 10.3389/fonc.2026.1752091

**Published:** 2026-04-27

**Authors:** Rola H. Ali, Hind S. Al-Otaibi, Samer A. K. Abdulmoneim, Eiman M. A. Mohammed, Ahmad R. Alsaber, Abdulaziz Hassan, Nisreen Khalifa, Abdullah A. Ali, Amir A. Ahmed, Shakir Bahzad, Fahad G. Alenezi, Muath AlNassar, Abdulaziz AlJassim

**Affiliations:** 1 Department of Pathology, College of Medicine, Kuwait University, Jabriya, Kuwait; 2 Department of Histopathology, Al Sabah Hospital, Shuwaikh, Kuwait; 3 Department of Surgical Oncology, Kuwait Cancer Control Center, Shuwaikh, Kuwait; 4 Department of Surgery, Wad Madani Teaching Hospital, Wad Madani, Gezira, Sudan; 5 Molecular Genetics Laboratory, Kuwait Cancer Control Center, Shuwaikh, Kuwait; 6 Department of Management, College of Business and Economics, American University of Kuwait, Salmiya, Kuwait; 7 Department of Diagnostic Radiology, Jaber Alahmad Hospital, South Surra, Kuwait; 8 Department of Pediatric Oncology, NBK Children’s Hospital, Shuwaikh, Kuwait; 9 Department of Medical Oncology, Kuwait Cancer Control Center, Shuwaikh, Kuwait

**Keywords:** Ewing sarcoma, Ewing-like, extraskeletal, FET-ETS, RNA sequencing, YWHAE-NUTM2B

## Abstract

**Background:**

FET::ETS-rearranged Ewing sarcoma (ES) and non-Ewing undifferentiated small round-cell sarcomas (SRCSs) comprise a morphologically overlapping yet molecularly diverse group of aggressive sarcomas. They differ in genotype, anatomic distribution, age profile, and clinical behavior. Clarifying genotype–anatomic correlations—particularly distinctions between ES and non-Ewing SRCSs, as well as between skeletal and extraskeletal presentations within the Ewing group—is essential for accurate diagnosis and treatment planning, especially in morphologically ambiguous cases.

**Methods:**

Ninety undifferentiated SRCSs of bone and soft tissue diagnosed between 2016 and 2025 at a tertiary cancer center were retrospectively reviewed. Clinical, histopathologic, and molecular data were analyzed using targeted RNA next-generation sequencing (Archer FusionPlex Sarcoma Panel) and break-apart fluorescence *in situ* hybridization (FISH) for *EWSR1* and *FUS* rearrangements. ES (FET::ETS-rearranged SRCSs) and non-Ewing SRCSs were compared across skeletal and extraskeletal sites using univariate analysis, and survival outcomes were assessed.

**Results:**

Among 90 cases (47 males, 43 females; median age 18.5 years), 76 (84%) harbored FET::ETS fusions and/or *EWSR1/FUS* rearrangements on FISH, consistent with the ES family in the appropriate morphologic context. Of 44 fusion-confirmed tumors, *EWSR1::FLI1* predominated (39/44; 89%), followed by *EWSR1::ERG* (4/44; 9%) and *FUS::ERG* (1/44; 2%). Fourteen tumors (16%) were classified as non-Ewing SRCSs, including *CIC::DUX4* (n=5), *BCOR*-altered (n=1), *EWSR1::ATF1* (n=1), *EWSR1::CREB1* (n=1), *YWHAE::NUTM2B* (n=1), and five fusion-negative SRCSs. ESs peaked during adolescence and showed a strong osseous predilection (48/76; 63%), particularly within the axial skeleton (*p* < 0.001). Extraskeletal and visceral ESs occurred in relatively older patients. Although ES still represented most extraskeletal tumors (28/40; 70%), non-Ewing SRCSs were significantly more often extraskeletal (*p* < 0.001) and demonstrated a broader age distribution, including infantile cases. Skeletal versus extraskeletal presentation had no significant impact on stage, progression, or overall survival. In contrast, metastatic disease at presentation was the strongest adverse prognostic factor (*p* = 0.007).

**Conclusion:**

ES and non-Ewing SRCSs exhibit morphologic overlap but differ in genotype, anatomic distribution, and age pattern. Within the Ewing group, skeletal versus extraskeletal presentation did not influence outcome, despite clear age differences. Integrating molecular diagnostics with site-specific and histopathologic assessment enhances diagnostic accuracy and guides management of these biologically diverse tumors.

## 1 Introduction

Undifferentiated small round-cell sarcomas (SRCSs) of bone and soft tissue represent a rare and diagnostically challenging group of malignant neoplasms with overlapping morphology but substantial biologic and genetic diversity (1). Ewing sarcoma (ES), the most common and prototypic entity, is itself a rare sarcoma, with an annual incidence of one to three cases per million (2). ES is defined by a translocation fusing a FET family RNA-binding gene with an ETS transcription factor gene, most often *EWSR1::FLI1* derived from t(11;22)(q24;q12). Alternative ETS partners occur in fewer than 10% of ESs and include *ERG* (3), *FEV* (4, 5), and *ETV1/ETV4* (6, 7).

Advances in molecular profiling have uncovered a broader spectrum of SRCSs lacking canonical FET::ETS fusions. These tumors, collectively referred to as “Ewing-like” SRCSs, are heterogeneous lesions unified primarily by morphologic resemblance to ES (8). They differ in genetic profile, age distribution, anatomic predilection, and clinical outcome, and are categorized into three major groups in the 2020 WHO Classification of Soft Tissue and Bone Tumors (9). The first includes *EWSR1*-non-ETS fusion sarcomas, with partners such as *PATZ1* (10–12), *NFATC2* (13, 14), *SP3* (15), and *SMARCA5* (16). The second comprises *CIC*-rearranged sarcomas, most commonly *CIC::DUX4*, representing the predominant abnormality among *EWSR1*-negative SRCSs (17, 18). The third encompasses sarcomas with *BCOR* alterations, most often *BCOR::CCNB3* or internal tandem duplication (*BCOR*-ITD) (19, 20). Additional emerging and morphologically overlapping entities that fall outside these categories continue to be recognized, including undifferentiated SRCSs with *YWHAE* fusions and epithelioid/SRCSs with *EWSR1/FUS::CREB (ATF1/CREM)* fusions (20–22).

Fusion subtype correlates with age, anatomic site, and overall survival (23). ES arising in extraskeletal (24, 25) or visceral sites (26, 27) poses particular diagnostic challenges, especially in older adults (28). In these atypical settings—outside the classic young age and intraosseous context—ES may mimic other small round-cell neoplasms, including epithelial malignancies. Challenges are amplified in small biopsies or when immunohistochemistry is equivocal, emphasizing the importance of integrated molecular testing for diagnostic accuracy.

Accurate subclassification of SRCSs is essential, as therapeutic and prognostic frameworks increasingly rely on integrated molecular profiling to capture their biological diversity (29). Correlating molecular subtypes with anatomic and clinicopathologic features further enhances diagnostic precision and guides management—an imperative in regions where molecular diagnostics are newly established and institutional datasets remain limited.

Herein, we retrospectively analyzed 90 SRCSs of bone and soft tissue diagnosed between 2016 and 2025 at a tertiary cancer center. The objectives were to (i) define the molecular spectrum of ES and non-Ewing undifferentiated SRCSs at our institution during that period, (ii) explore genotype–anatomic correlations, and (iii) assess clinicopathologic and survival profiles.

## 2 Materials and methods

### 2.1 Case selection

Cases of molecularly tested undifferentiated SRCSs were retrospectively identified from the database at the Molecular Genetics Lab, Kuwait Cancer Center (2016–2025). Inclusion criteria comprised: (1) canonical ES harboring FET::ETS fusions; (2) WHO-defined undifferentiated SRCSs with alternative fusions (*EWSR1*–non-ETS fusion sarcomas, *CIC*-rearranged sarcomas, and *BCOR*-altered sarcomas); (3) emerging molecularly defined SRCSs not currently included in the WHO classification; and (4) fusion-negative undifferentiated SRCSs, not otherwise specified (NOS). In this study, the term ‘non-Ewing SRCS’ is used descriptively to refer to undifferentiated SRCSs lacking canonical FET::ETS fusions and is not intended to correspond strictly to WHO-defined ‘Ewing-like’ categories. Cases with failed molecular testing were excluded.

Clinicopathological data (age, sex, primary site, tumor size, stage at diagnosis [localized vs metastatic], treatment details, and status at last contact) were retrieved from medical records; follow-up data were included where available. Hematoxylin- and eosin-stained (H&E) slides were reviewed by a pathologist (RHA) for growth pattern, stroma, cytomorphology, nuclear features, necrosis, and mitotic activity. Immunohistochemical studies were performed using validated protocols; however, detailed documentation of antibody clones and dilutions was not uniformly available in this retrospective cohort. Commonly assessed markers included CD99, NKX2.2, S100, cytokeratin, and WT1, while additional markers varied based on the differential diagnosis.

Preoperative imaging was reviewed to determine anatomic origin, and tumors were categorized as either skeletal (axial, appendicular, or craniofacial) or extraskeletal (soft tissues of trunk/limbs or visceral sites). Lesions centered in medullary cavity and/or cortex of bone, with or without soft-tissue extension, were designated skeletal. Tumors arising primarily in deep or superficial soft tissues or visceral organs were classified as extraskeletal, including those with secondary bone invasion.

### 2.2 Targeted RNA sequencing

Formalin-fixed, paraffin-embedded (FFPE) tumor samples from 62 cases were submitted for targeted RNA sequencing after a provisional diagnosis of ES or undifferentiated SRCS based on morphology and immunophenotype. Sequencing and bioinformatics analyses were performed in-house at the Molecular Genetics Laboratory, Kuwait Cancer Center.

Total RNA was extracted using the RecoverAll Total Nucleic Acid Isolation Kit (Thermo Fisher Scientific), quantified on a Qubit 3.0 Fluorometer (Thermo Fisher Scientific, USA), and assessed for integrity using the Agilent TapeStation system (Agilent Technologies, USA). RNA quality was further evaluated with the Archer PreSeq RNA QC Assay (ArcherDX, Boulder, CO, USA); samples with a cycle threshold (Ct) >28 were considered degraded and excluded. Whenever available, 200 ng of unsheared RNA was considered ideal, although samples with ≥50 ng were also analyzed.

Libraries were prepared using the Archer FusionPlex Sarcoma Panel (v2; ArcherDX) according to manufacturer instructions. The Anchored Multiplex PCR (AMP™) approach uses unidirectional gene-specific primers to capture both known and novel fusions (30). Libraries were constructed using the Archer Universal RNA Reagent Kit, which incorporates molecular barcode adapters for unique molecule tagging, enabling deduplication and high-confidence fusion calling. The panel targets 62 genes across 659 primer pairs implicated in sarcoma-related chromosomal rearrangements. Sequencing was performed on the Ion Torrent S5 XL platform.

Data were analyzed using Archer Analysis software (v5.0.4). High-confidence fusions required ≥5 unique breakpoint-spanning reads and ≥3 reads with distinct start sites. Analytical sensitivity was optimized for canonical oncogenic isoforms but could be reduced in samples with <10% tumor cellularity or poor RNA quality (<10 unique start-site reads at control loci or <40% RNA reads). Internal QC metrics (mapped reads, duplication rate) were reviewed to ensure adequacy.

### 2.3 Fluorescence *in situ* hybridization

*EWSR1* (22q12) and *FUS* (16p11) rearrangements were assessed in 87 FFPE sections using locus-specific dual-color break-apart probes (Abbott Molecular/Vysis). The 5′ (centromeric) probe was Spectrum Orange and the 3′ (telomeric) probe Spectrum Green.

Paraffin sections (4 µm) were mounted on poly-L-lysine slides, baked overnight, deparaffinized, and dehydrated in ethanol. Pretreatment included heat incubation in sodium citrate buffer, protease digestion, and final ethanol dehydration. Probe (≈20 µL) was applied and slides sealed with rubber cement. Co-denaturation was performed at 85 °C for 5 min, followed by overnight hybridization at 37 °C (ThermoBrite, Abbott Molecular, USA). After washing in 2× SSC/0.3% NP-40 at 72 °C, slides were counterstained with DAPI.

Tumor-rich areas were identified on the FISH slides corresponding to that on H&E. Hybridization signals were scored in 100 non-overlapping nuclei on a Zeiss fluorescence microscope (Carl Zeiss, Jena, Germany) using oil immersion and appropriate filters. Normal nuclei showed two fused (yellow) signals; rearranged nuclei typically showed one fused and one split (orange–green) pattern. Atypical or mixed patterns were also noted. A split was defined as separation exceeding twice the signal diameter. A cutoff of ≥20% split-signals defined positivity.

### 2.4 Statistical analysis

Descriptive statistics were used for central tendency (mean, median), dispersion (range, standard deviation), and frequency distributions, supplemented by graphical visualization. ES versus non-Ewing SRCSs and skeletal versus extraskeletal lesions were compared using univariate analyses. Pearson’s chi-squared test was applied to categorical variables (e.g., anatomic site, fusion category, stage), and independent two-sample t-tests to continuous variables (e.g., tumor size, age). Survival was assessed using Kaplan–Meier methodology. Overall survival (OS) was defined from date of diagnosis to death or last follow-up; progression-free survival (PFS) from diagnosis to first radiologic/clinical progression or recurrence. Patients without events were censored at last contact. All statistical analyses were conducted in JAMOVI (v2.5.7.0), with two-tailed significance set at *p* < 0.05.

## 3 Results

### 3.1 Overall cohort description

Between 2016 and 2025, a total of 90 cases of undifferentiated SRCSs of bone and soft tissue were identified, all of which underwent FISH and/or Archer NGS analysis with interpretable results. Clinicopathological features are summarized in **Table 1**.

**Table 1:** Clinicopathological characteristics.

Characteristics	n (%)
Age	
Mean (SD)	22.1 (14.2)
Range	0.0 - 69.7
Age groups (in decades)	
0 to 9	17 (18.9%)
10 to 19	30 (33.3%)
20 to 29	21 (23.3%)
30 to 39	10 (11.1%)
≥40	12 (13.3%)
Age groups (binary)	
≤18	45 (50.0%)
>18	45 (50.0%)
Gender	
Female	43 (47.8%)
Male	47 (52.2%)
Tumor size (cm)	
Mean (SD)	9.4 (5.1)
Range	1.4 - 29.0
Size category	
<8 cm	39 (44.8%)
≥8 cm	48 (55.2%)
Overall anatomical region	
Trunk	42 (46.7%)
Extremity	23 (25.6%)
Limb girdle	16 (17.8%)
Acral	4 (4.4%)
Head & neck	5 (5.6%)
Skeletal vs extraskeletal	
Skeletal	50 (55.6%)
Axial	29 (58.0%)
Appendicular	17 (34.0%)
Acral	3 (6.0%)
Craniofacial	1 (2.0%)
Extra-skeletal	40 (44.4%)
Deep soft tissue	14 (35.0%)
Subcutaneous/superficial	11 (27.5%)
Visceral/retroperitoneal	11 (27.5%)
Head & neck	4 (10.0%)
Histological/molecular categories	
FET::ETS–rearranged Ewing sarcoma	76 (84.4%)
Non-Ewing small round-cell sarcomas	14 (15.6%)
Baseline stage at diagnosis	
Localized	62 (68.9%)
Metastatic	24 (26.7%)
Unknown	4 (4.4%)
Total	90

**Figure 1:**
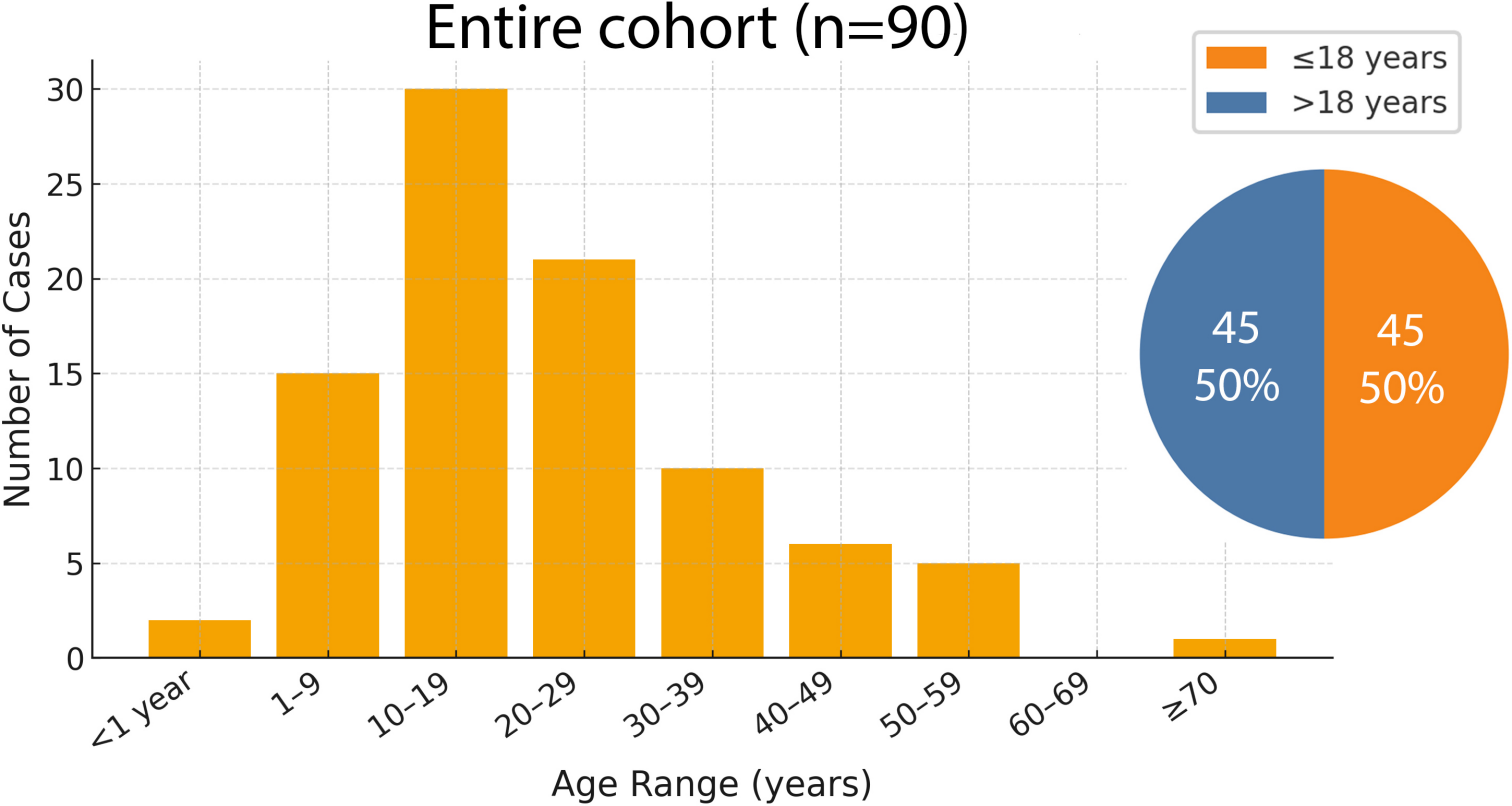
Age distribution of the entire small round-cell sarcoma cohort (n = 90) showing a predominance in the second decade of life, with pediatric (≤18 years) and adult (>18 years) patients equally represented.

Pediatric (≤18 years) and adult (>18 years) patients were equally represented (45 each), with 43 females (48%) and 47 males (52%). Age at diagnosis ranged from 1 week to 69.7 years (median 18.5; mean 22.1 ± 14.2). As shown in **Figure 1**, the largest proportion of tumors occurred in adolescents (10–19 years, 33.3%), with a peak during mid- to late adolescence (15–19 years; 20%). Only 13.3% occurred at ≥40 years.

In terms of anatomical distribution, 50 tumors (55.6%) were skeletal, while 40 (44.4%) occurred in extraskeletal somatic soft-tissue or visceral locations. Within skeletal tumors, the axial skeleton was most frequently involved (29/50; 58%), followed by appendicular sites (17/50; 34%), with fewer acral (3/50; 6%) and craniofacial (1/50; 2%) cases. Extraskeletal tumors most commonly arose in deep soft tissue (14/40; 35%), subcutaneous/superficial tissue (11/40; 27.5%), and visceral or retroperitoneal locations (11/40; 27.5%), with fewer arising in head and neck regions (4/40; 10%).

### 3.2 Molecular and FISH findings

**Figure 2:**
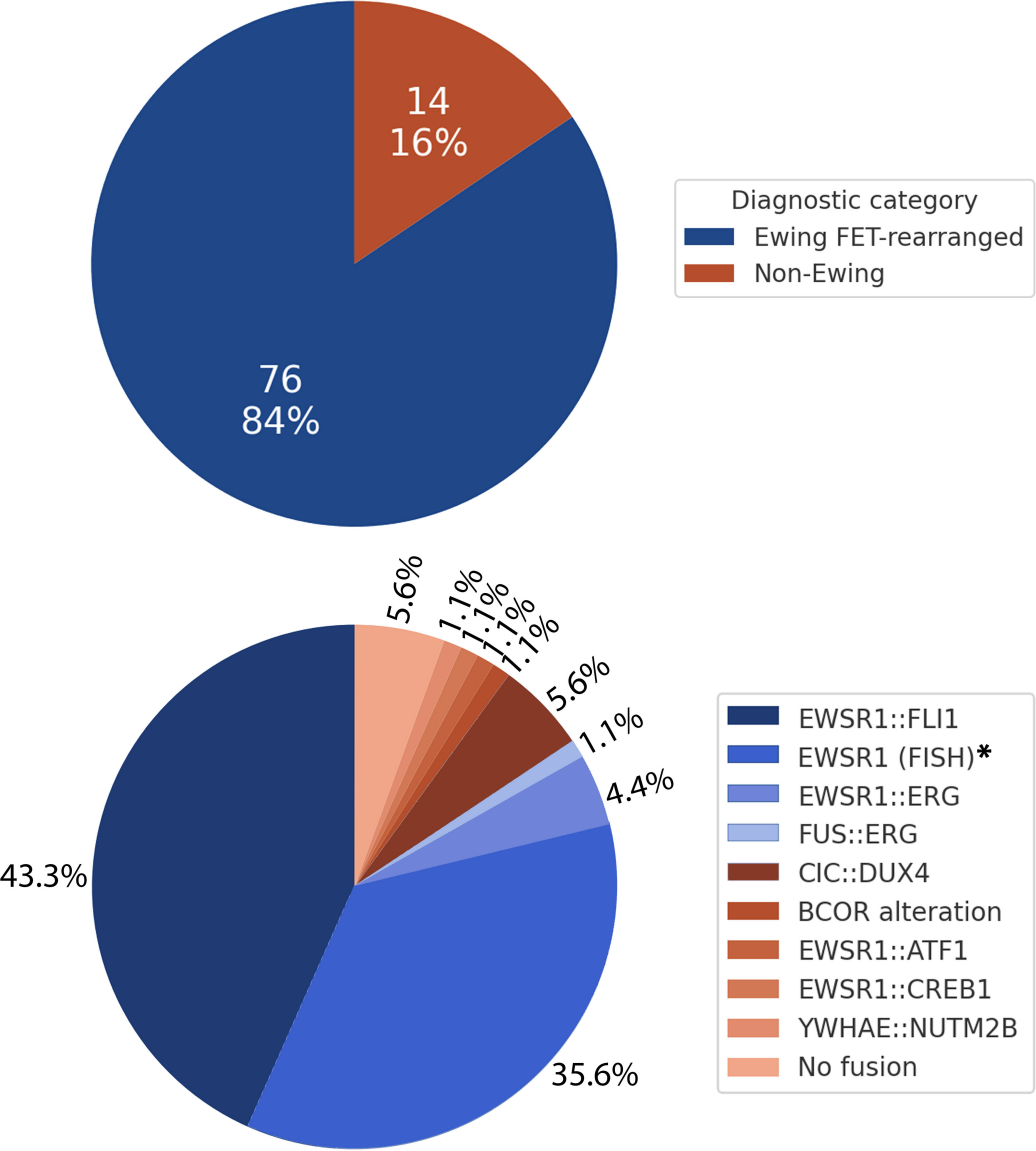
Molecular classification of the study cohort (n=90). The upper pie chart illustrates the proportion of FET::ETS-rearranged (Ewing family) versus non-Ewing small round-cell sarcomas. The lower chart depicts specific molecular subgroups. *EWSR1 (FISH) = positive *EWSR1* break-apart FISH without sequencing data.

Among 87 tumors tested by FISH, 72/87 (83%) demonstrated *EWSR1* and/or *FUS* rearrangement. RNA sequencing in 62 cases detected an in-frame fusion transcript in 53/62 (86%). Combined molecular approaches established a definitive fusion in 59/90 (66%) cases, enabling broad classification into FET::ETS-rearranged Ewing family and non-Ewing SRCSs with alternative or absent fusions (**Figure 2**).

A diagnosis within the Ewing family was made in 76/90 cases (84%) based on characteristic morphology and molecular confirmation. Of these, 44 (58%) were fusion-confirmed by sequencing (± FISH), and 32 (42%) showed *EWSR1* rearrangement by FISH alone. Although break-apart FISH does not identify fusion partners, these cases were classified as ES when morphology and immunophenotype were classical. Concordance between sequencing and FISH was 85% (40/47); discordance (7 cases, 15%) largely reflected technical limitations due to scant or necrotic tissue.

**Figure 3:**
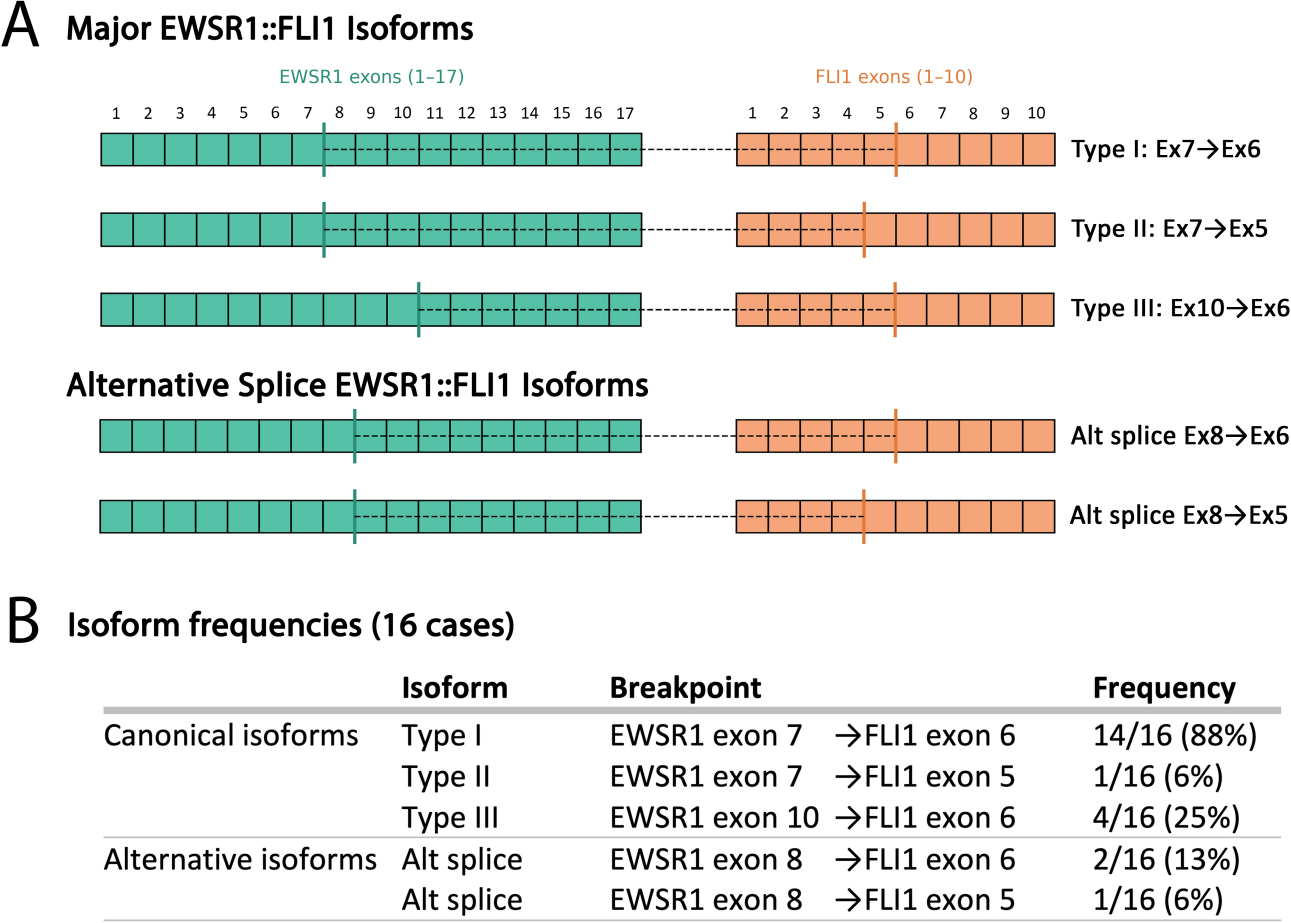
Schematic representation of *EWSR1::FLI1* RNA fusion isoforms in 16 Ewing sarcomas. **(A)** Major/canonical (Type I–III) isoforms and alternative splice junctions. **(B)** Frequency distribution among the cases.

Among fusion-confirmed ESs, the predominant rearrangement was *EWSR1::FLI1* (39/44; 89%), followed by *EWSR1::ERG* (4/44; 9%) and *FUS::ERG* (1/44; 2%). Transcript-level data in 16 *EWSR1::FLI1* cases showed mostly canonical isoforms from t(11;22)(q24;q12): Type I (*EWSR1* exon 7–*FLI1* exon 6; 88%), Type III (exon 10–exon 6; 25%), and Type II (exon 7–exon 5; 6%). Occasional minor alternative splice products were identified reflecting recognized transcript variability (**Figure 3**).

Non-Ewing molecular alterations were identified in 9/90 cases (10%): *CIC::DUX4* (n=5), *BCOR* alteration (n=1), *EWSR1::ATF1* (n=1), *EWSR1::CREB1* (n=1), and *YWHAE::NUTM2B* (n=1). Five additional cases (6%) were fusion-negative and classified as undifferentiated round-cell sarcoma, not otherwise specified (NOS).

### 3.3 Clinicopathologic patterns across Ewing sarcoma and mimics

**Figure 4:**
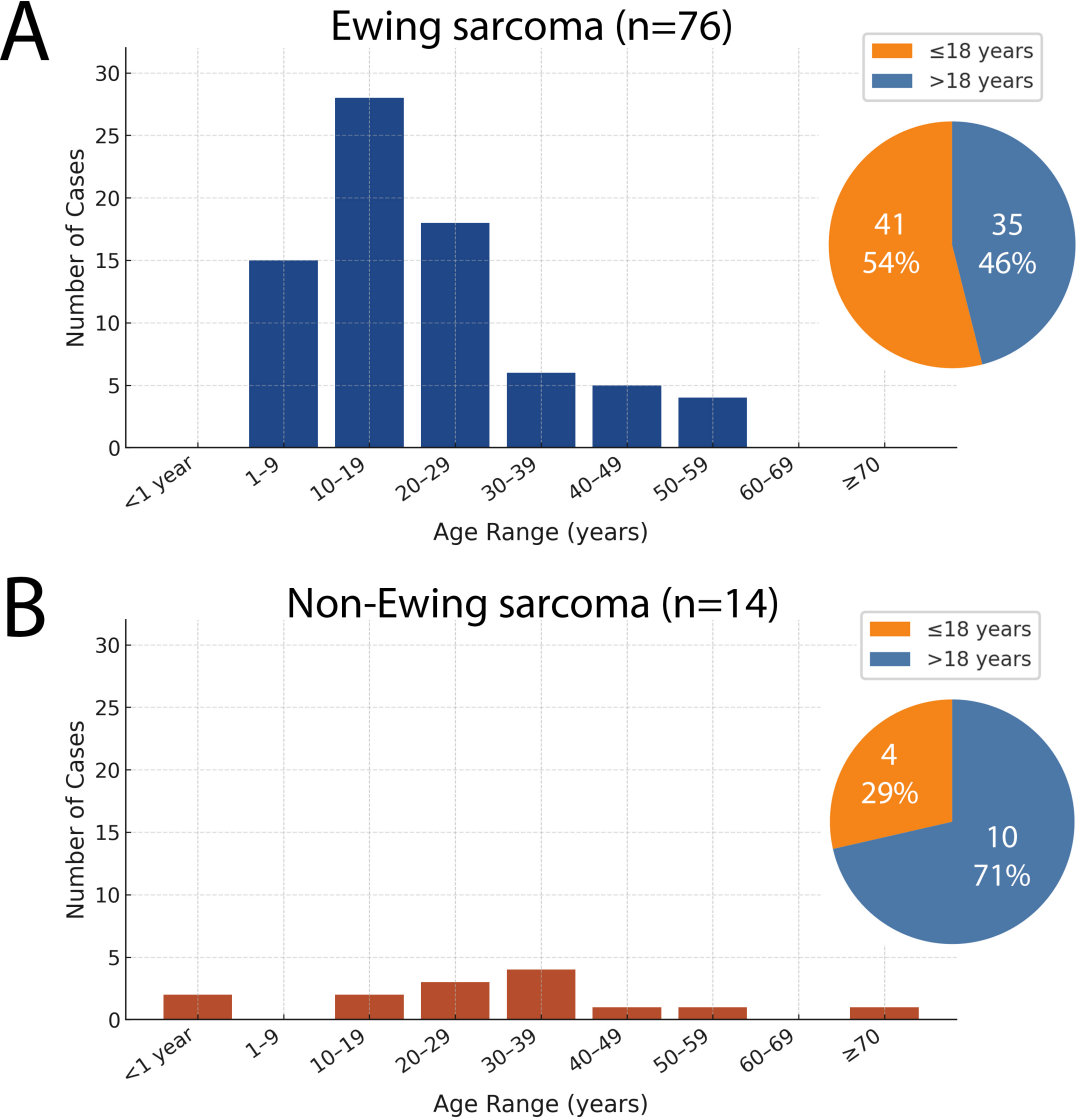
Age distribution of FET::ETS-rearranged Ewing-family vs non-Ewing small round-cell sarcomas. **(A)** Ewing group showing adolescent peak. **(B)** Non-Ewing group showing broader age range with infantile cases and predominance in adults (>18 years).

Age distribution differed between the diagnostic groups. ES family (FET::ETS-rearranged SRCSs) showed the expected adolescent/young-adult peak (54% ≤18 years, 46% >18). In contrast, non-Ewing SRCSs, with alternative or absent gene fusions, spanned a broader age range, including two infantile cases, with most (71%) occurring in adults (**Figure 4**).

**Figure 5:**
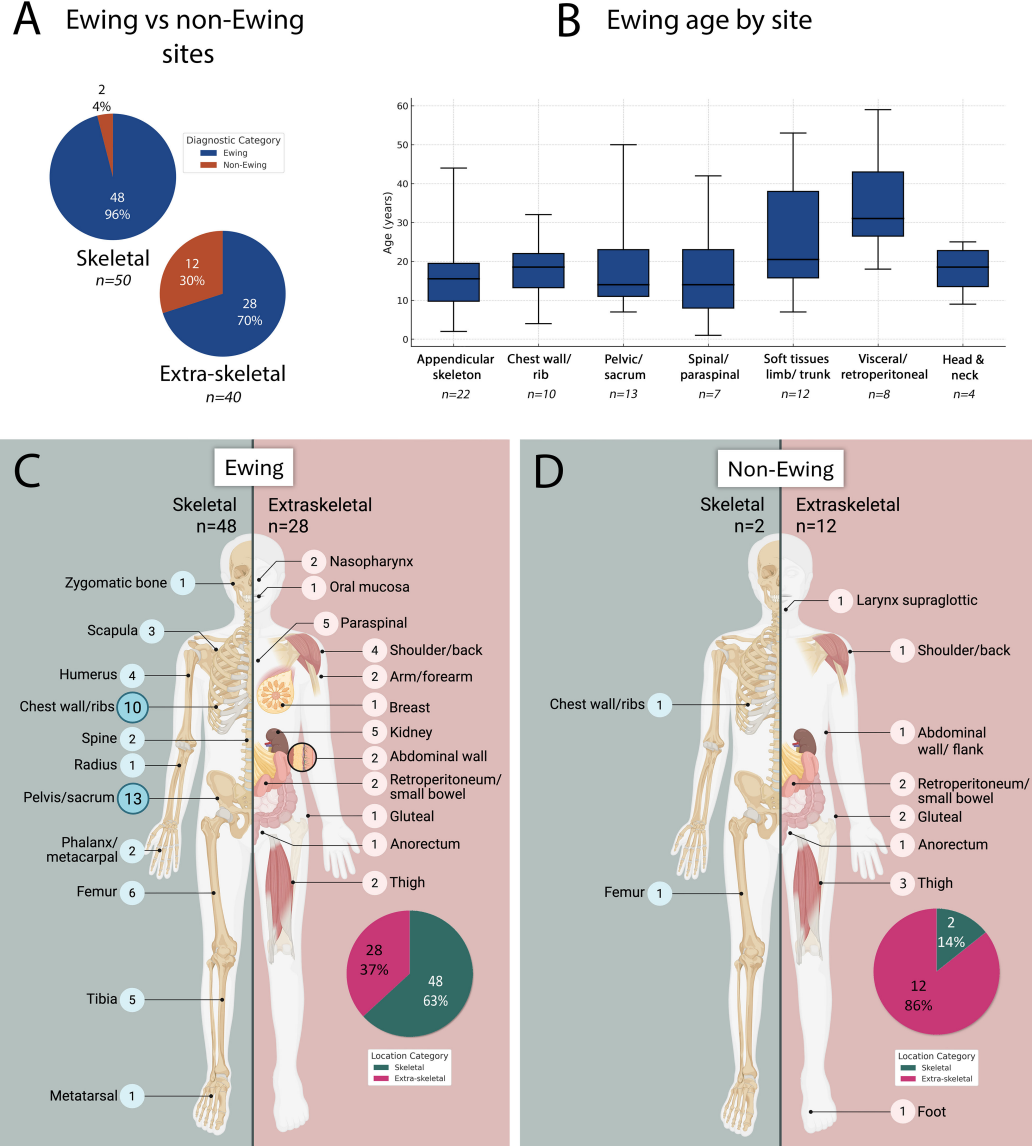
Anatomic distribution of FET::ETS-rearranged Ewing vs non-Ewing small round-cell sarcomas. **(A)** Skeletal predominance of Ewing sarcoma. **(B)** Boxplot showing older median age for extraskeletal Ewing cases. **(C)** Subsites of Ewing cases. **(D)** Subsites of non-Ewing cases. Created in BioRender. Ali, (R) (2025) https://BioRender.com.

ES demonstrated a strong osseous predilection, arising in bone in 48/76 (63%) compared with 2/14 (14%) ES mimics (*p* < 0.001), consistent with its well-recognized skeletal predominance. Within the skeletal subset, ES accounted for 96% of tumors (*p* < 0.001). Anatomically, skeletal ES most often involved the axial skeleton (28/48; 58%), particularly the pelvis (n=13) and chest wall/ribs (n=10), followed by scapula (n=3) and spine (n=2). Nineteen cases involved appendicular bones, and one was craniofacial. Extraskeletal ES occurred across a range of sites, including deep somatic soft tissue (n=9), superficial/subcutaneous tissue (n=8), visceral/retroperitoneal locations (n=8), and head/neck (n=3). ES arising in soft tissue and visceral sites tended to present at older ages (**Figure 5**), with a mean age of 26.2 years (range 1–59; median 21.5), increasing to a mean of 32 years (range 18–59; median 31) for visceral cases.

**Figure 6:**
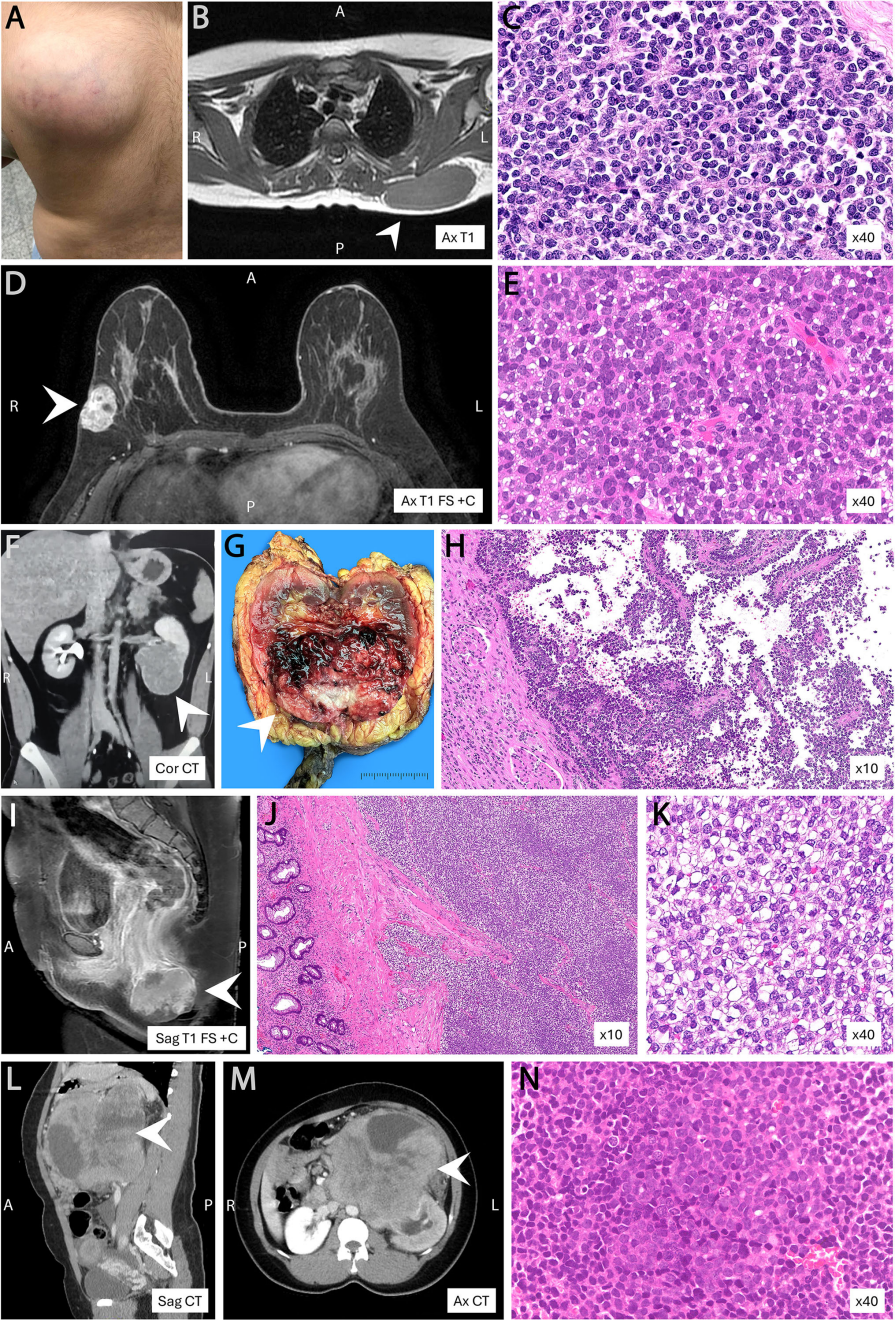
Radiologic–pathologic correlation in extraskeletal FET::ETS-rearranged Ewing sarcomas. **(A–C)** Subcutaneous Ewing on the back with rosette-forming small round cells. **(D, E)** Breast lesion mimicking primary solid-papillary carcinoma. **(F–H)** Renal tumor with perivascular pseudopapillary architecture simulating renal carcinoma. **(I–K)** Anorectal tumor with clear-cell change infiltrating the bowel wall, resembling metastatic carcinoma. **(L–N)** Retroperitoneal mass abutting pancreas, kidney, and bowel, showing classic Ewing morphology. Abbreviations: A, anterior; P, posterior; R, right; L, left; Ax, axial; Cor, coronal; Sag, sagittal; T1, T1-weighted MRI; T1 FS +C, fat-saturated T1 post-contrast MRI; CT, computed tomography.

Visceral/retroperitoneal ES was identified in 8/76 (10.5%) cases, including renal (n=5), small-intestinal (n=1), anorectal (n=1), and primary retroperitoneal (n=1) tumors. Most harbored *EWSR1::FLI1* fusions, with one *EWSR1::ERG*. Renal ES (n=5) occurred in adults (range 18–59; median 40), were large (mean 11.0 cm; range 6.5–17.0), and frequently demonstrated renal sinus/perinephric fat invasion (4/5; 80%) and renal vein/IVC tumor thrombus (4/5; 80%), in keeping with their angioinvasive behavior. Extraskeletal soft-tissue and visceral ES often posed diagnostic and therapeutic challenges due to atypical presentations (**Figure 6**), with superficial lesions occasionally misinterpreted as benign clinically leading to unplanned marginal excisions. Visceral tumors—particularly those with atypical cytologic features (enlarged nuclei, irregular contours, prominent nucleoli)—overlapped with small-cell carcinoma and other tumor mimics, necessitating broad immunohistochemical and molecular evaluation. Histologically, ES typically formed solid sheets of uniform small round cells, with rosettes in 27.6% (21/76), yet occasionally showed alternative architectures, including nests/trabeculae (21%; 16/76), pseudopapillary (7.9%; 6/76), and rarely multicystic or pseudoglandular patterns (2.6%; 2/76). Most were matrix-poor (80.3%; 61/76) with scant stroma. Immunohistochemically, diffuse CD99 and NKX2.2 expression was characteristic (*p* < 0.001), while focal/patchy cytokeratin (25%) and S100 (21%) expression were recognized diagnostic pitfalls.

**Figure 7:**
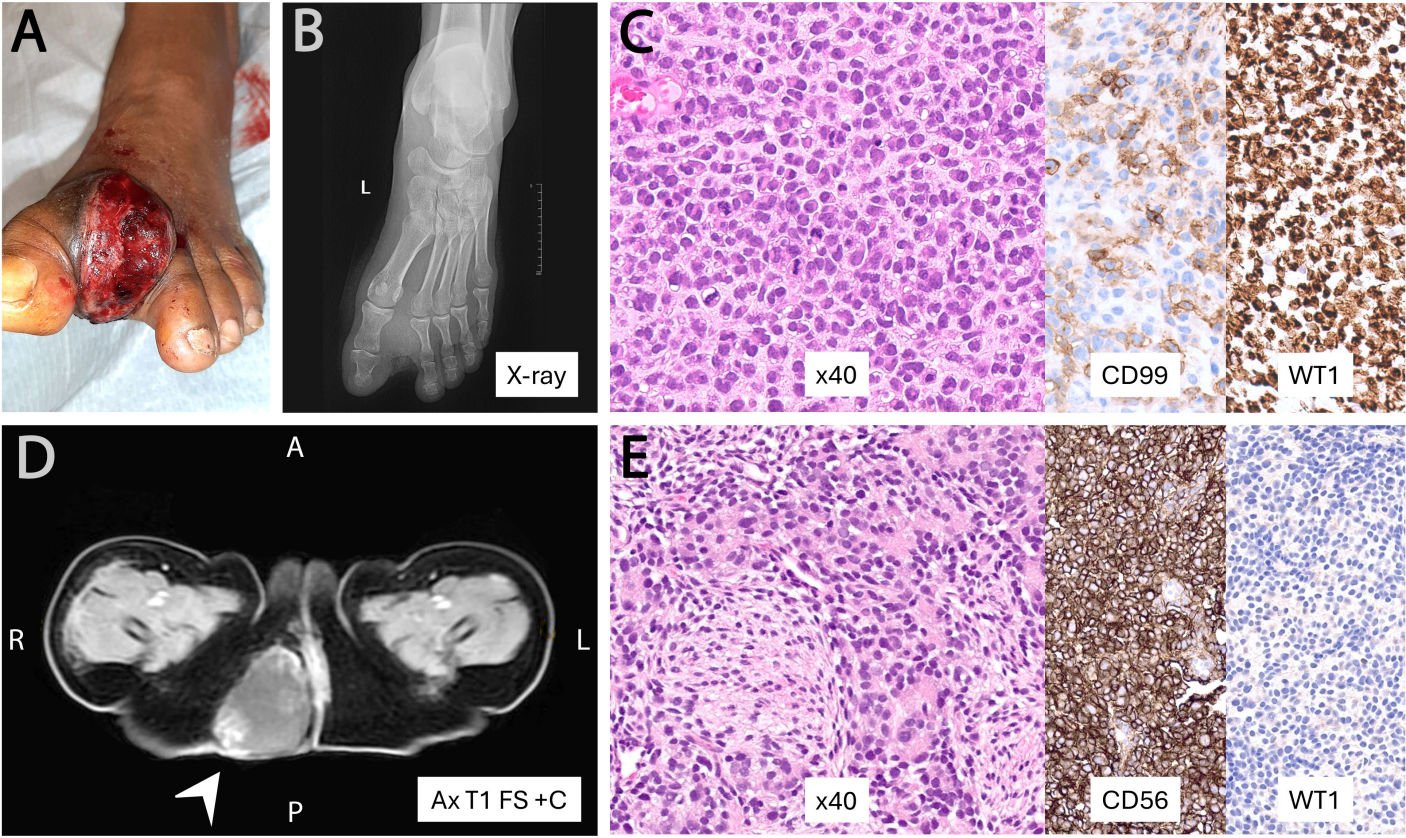
Representative fusion-positive non-Ewing small round-cell sarcomas. **(A–C)** *CIC::DUX4* sarcoma of the foot, ulcerated and clinically mimicking epithelioid sarcoma, composed of round-to-epithelioid cells with brisk mitoses, CD99 focal and WT1 diffuse. **(D, E)** *YWHAE::NUTM2B* sarcoma of the gluteal region in a neonate, showing primitive round-to-spindle morphology with CD56 positivity and WT1 negativity. Abbreviations: A, anterior; P, posterior; R, right; L, left; Ax T1 FS +C, axial fat-saturated T1-weighted MRI post-contrast.

**Figure 8:**
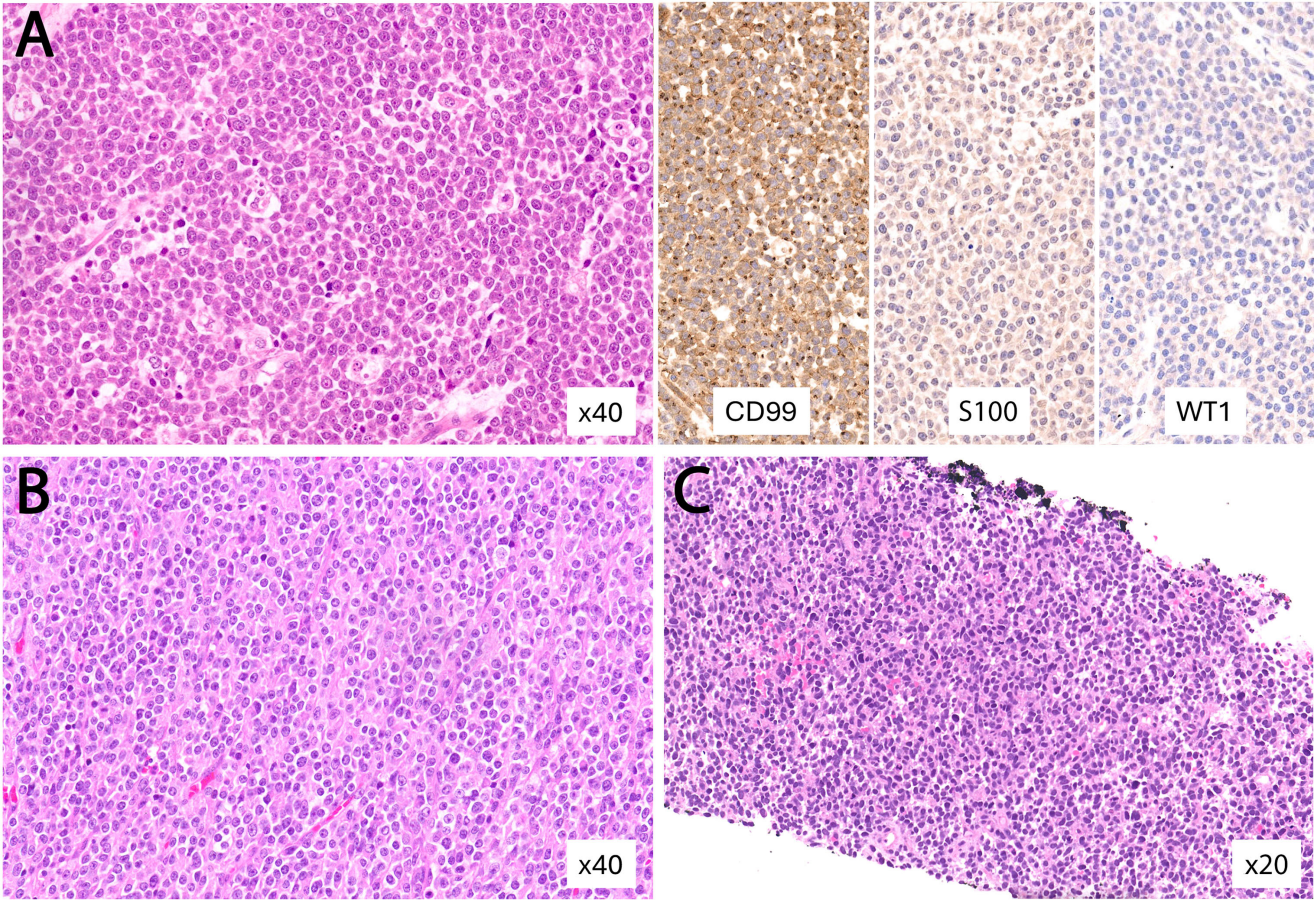
Representative fusion-negative undifferentiated small round-cell sarcomas, not otherwise specified (NOS). **(A)** Metastatic sarcoma to the lung from a previously amputated lower limb, composed of sheets of round to ovoid cells with vesicular nuclei and prominent nucleoli. The immunoprofile is nonspecific, showing dot-like CD99 positivity and negativity for an extensive immunopanel, including S100 and WT1. **(B)** Extraskeletal soft tissue mass of the back composed of round cells with irregular vesicular nuclei and focally prominent nucleoli. **(C)** Core biopsy of an anorectal mass showing small round to epithelioid cells with a broad differential diagnosis, including carcinoma, lymphoma, and fusion-positive sarcoma; the immunostaining panel was nonspecific.

Non-Ewing SRCSs showed a predominantly extraskeletal distribution (12/14; 86%) (**Figure 5**), including 3/12 (21.4%) with visceral involvement: one *CIC*-rearranged sarcoma, one *EWSR1::CREB1*-positive malignant gastrointestinal neuroectodermal tumor (GNET), and one fusion-negative undifferentiated SRCS (NOS) centered in the anorectum. The *EWSR1::CREB1*-positive GNET case is included as a diagnostic mimicker encountered on limited biopsy and is not considered a true SRCS. Representative non-Ewing SRCSs are shown in **Figures 7**, **8**, and their clinicopathologic features are summarized in **Table 2**. Histologically, the non-Ewing cases demonstrated greater morphologic and immunophenotypic heterogeneity, reflecting their underlying genetic diversity; for example, WT1 expression was characteristic of *CIC*-rearranged sarcomas (*p* < 0.001). In the fusion-negative cases, an extensive immunohistochemical panel, including markers for epithelial, melanocytic, lymphoid, myogenic, vascular, and neuroendocrine differentiation, failed to demonstrate specific lineage differentiation, supporting an exclusion diagnosis of undifferentiated SRCS, NOS.

**Table 2:** Contrasting Ewing and non-Ewing undifferentiated small round-cell sarcomas.

Variable	Ewing n = 76	Non-Ewing n = 14	Total n = 90	p-value
Age group				0.081
>18	35 (46.1%)	10 (71.4%)	45 (50.0%)	
≤18	41 (53.9%)	4 (28.6%)	45 (50.0%)	
Gender				0.688
Female	37 (48.7%)	6 (42.9%)	43 (47.8%)	
Male	39 (51.3%)	8 (57.1%)	47 (52.2%)	
Tumor size				0.099
≥8 cm	39 (51.3%)	9 (64.3%)	48 (53.3%)	
<8 cm	34 (44.7%)	5 (35.7%)	39 (43.3%)	
Unknown	3 (4.0%)	0 (0.0%)	3 (3.3%)	
Location				< 0.001
Extraskeletal	28 (36.8%)	12 (85.7%)	40 (44.4%)	
Skeletal	48 (63.2%)	2 (14.3%)	50 (55.6%)	
Baseline stage at diagnosis				0.209
Localized	55 (75.3%)	7 (53.8%)	62 (72.1%)	
Metastatic	18 (24.7%)	6 (46.2%)	24 (27.9%)	
Unknown	3	1	4	
Post-baseline progression				0.250
Yes	24 (33.8%)	7 (50.0%)	31 (36.5%)	
No	47 (66.2%)	7 (50.0%)	54 (63.5%)	
Unknown	5	0	5	
Neoadjuvant chemotherapy				0.017
Yes	56 (77.8%)	7 (70.0%)	63 (76.8%)	
No	16 (22.2%)	3 (30.0%)	19 (23.2%)	
Unknown	4	4	8	
Adjuvant chemotherapy				0.012
Yes	48 (66.7%)	5 (50.0%)	53 (64.6%)	
No	24 (33.3%)	5 (50.0%)	29 (35.4%)	
Unknown	4	4	8	
Definitive Surgery				0.039
Yes	45 (63.4%)	7 (70.0%)	52 (64.2%)	
No	26 (36.6%)	3 (30.0%)	29 (35.8%)	
Unknown	5	4	9	
Radiation				0.006
Yes	18 (25.4%)	3 (33.3%)	21 (26.2%)	
No	53 (74.6%)	6 (66.7%)	59 (73.8%)	
Unknown	5	5	10	
Status last contact				0.063
Dead	12 (16.9%)	3 (27.3%)	15 (18.3%)	
No evidence of disease	33 (46.5%)	1 (9.0%)	34 (41.5%)	
Progressive disease	7 (9.8%)	3 (27.3%)	10 (12.2%)	
Stable disease	19 (26.8%)	4 (36.4%)	23 (28.0%)	
Unknown	5	3	8	

### 3.4 Outcome analysis

**Figure 9:**
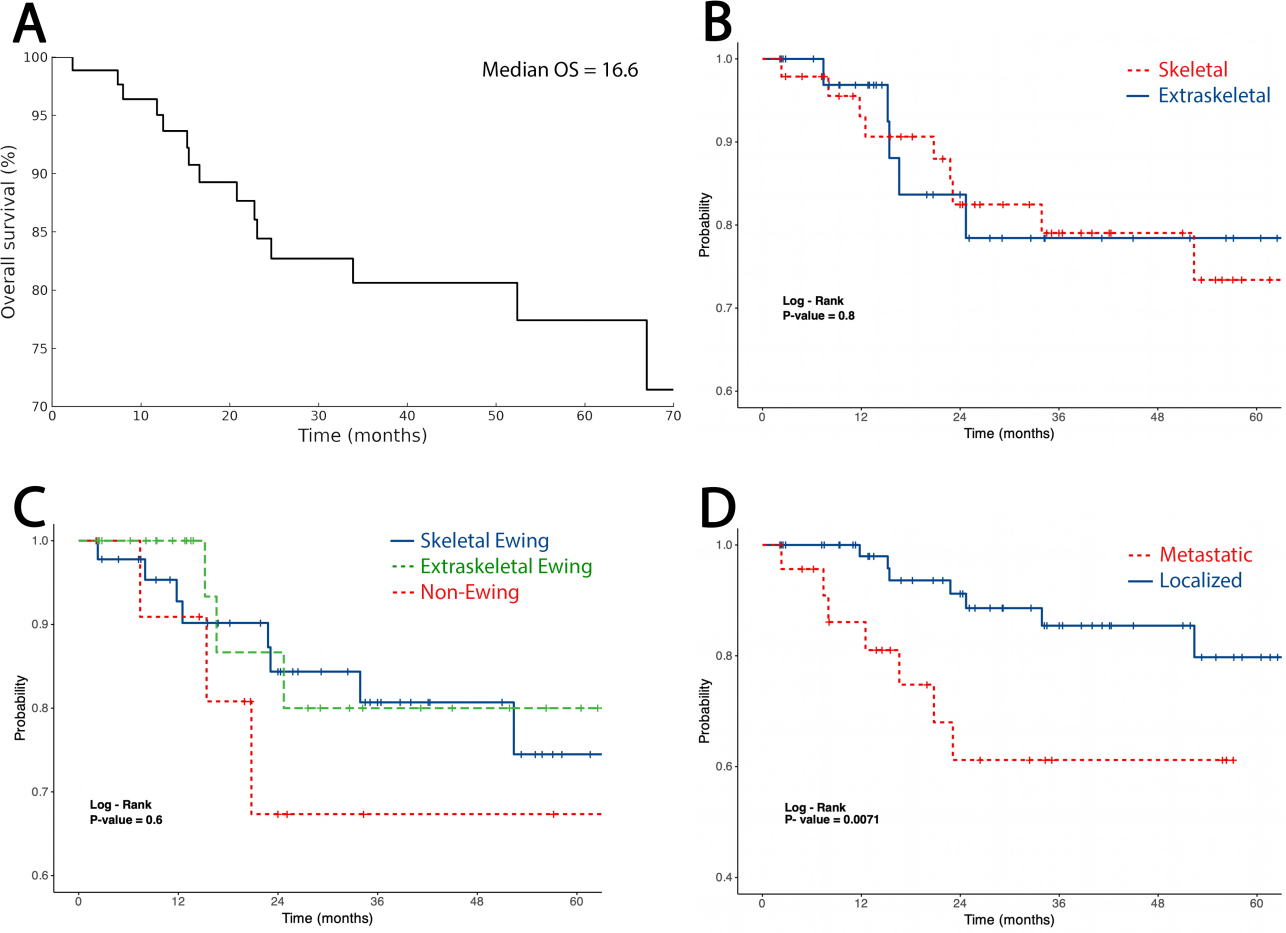
Kaplan–Meier overall survival analyses. **(A)** Overall survival (OS) of the cohort. **(B)** Skeletal vs. extraskeletal tumors showing no survival difference (*p* = 0.80). **(C)** Skeletal Ewing, extraskeletal Ewing, and non-Ewing sarcomas showing comparable outcomes (*p* = 0.60). **(D)** Localized vs. metastatic disease, with significantly poorer survival in metastatic cases (*p* = 0.007).

Seventy-one percent of patients presented with localized disease, whereas 29% were metastatic at diagnosis, most commonly to the lungs. Stage distribution did not differ significantly between ES and non-Ewing SRCSs (*p* = 0.33) or between skeletal and extraskeletal tumors (*p* = 1.00). Median follow-up was 25.1 months (range 2.1–223.7; 0.2–18.6 years). At last follow-up, 15 patients had died, yielding a median overall survival (OS) of 16.6 months (range 2.3–67.0 months). The estimated 2-year and 4-year OS rates were ~83% and 79%, respectively, reflecting substantial censoring beyond 2 years. Survival was comparable between skeletal and extraskeletal tumors and across diagnostic categories, while metastatic presentation conferred significantly inferior OS (*p* = 0.007) (**Figure 9**).

Overall, 31 patients experienced disease progression during therapy or follow-up, including 15 (48%) who had initially presented with localized disease. Among the localized group (n = 61), progression represented 24.6% (15/61) of cases: eight developed distant metastases, five isolated local recurrence, and two developed both local and distant progression. Median time to progression was 21.2 months (range 5.3–187.4). Metastatic presentation carried a markedly higher risk of progression than localized disease (16/25 vs 15/61; *p* < 0.001).

Among 33 patients who developed metastatic disease at any time, the lungs were most frequently involved (19/33, 57.6%), followed by lymph nodes (17/33, 51.5%), bone (11/33, 33.3%), and brain/skull (7/33, 21.2%). Overall metastatic risk did not differ between skeletal and extraskeletal tumors (*p* = 0.65). Visceral primaries, however, showed a markedly higher metastatic rate (72.7%; *p* = 0.004) with enrichment for lymph-node metastasis (54.5%; *p* < 0.001). Diagnostic category influenced metastatic behavior: non-Ewing SRCSs had higher metastatic rates (64.3%; *p* = 0.020) and more frequent lymph-node involvement (50%; *p* = 0.002) than ES.

Most patients received multimodal therapy (chemotherapy, surgery, and/or radiotherapy). Neoadjuvant and adjuvant chemotherapy were administered in 76.8% and 64.6% of evaluable cases, respectively, with VDC/IE (vincristine, doxorubicin, cyclophosphamide alternating with ifosfamide/etoposide) being the predominant regimen. Neoadjuvant chemotherapy was used more often in skeletal tumors (88%) than in extraskeletal tumors (48%) because the latter—particularly subcutaneous or visceral lesions—were more frequently excised upfront. Definitive surgery with or without radiotherapy was performed in 52/81 cases (64.2%). Radiotherapy served as the primary local-control modality in 26.2% when complete excision was not feasible (e.g., pelvic tumors).

## 4 Discussion

This retrospective study provides a clinicopathologic and molecular overview of undifferentiated SRCSs of bone and soft tissue—within and beyond the Ewing family—diagnosed over nearly a decade at our cancer center. To our knowledge, this represents the most comprehensively characterized cohort from our region, with integrated genotype–anatomic correlations across skeletal and extraskeletal SRCSs. Among 90 tumors, most (84%) harbored canonical FET::ETS fusions or *EWSR1/FUS* rearrangements consistent with ES, while the remaining 16% comprised molecularly distinct non-Ewing SRCSs. *EWSR1::FLI1* was the predominant fusion in ES, followed by *EWSR1::ERG* and rare *FUS::ERG* rearrangements. The non-Ewing SRCS subset included *CIC::DUX4*, *BCOR*-altered, *YWHAE::NUTM2B*, and *EWSR1::ATF1*-positive sarcomas, reflecting the expanding molecular spectrum of undifferentiated SRCSs (8, 20, 31–33). Notably, the *EWSR1::ATF1*-rearranged case in our cohort did not conform to any of the established WHO entities (i.e. clear cell sarcoma or angiomatoid fibrous histiocytoma) and was recently reported in a series of novel unclassified mesenchymal neoplasms characterized by epithelioid/round-cell morphology and *EWSR1::ATF1* fusion, not fitting any defined WHO category (22).

Consistent with prior work, ES in this cohort showed an osseous predilection, particularly in the axial skeleton, whereas non-Ewing counterparts more often arose in extraskeletal sites. Although grouped pragmatically, non-Ewing undifferentiated SRCSs remain biologically and anatomically heterogeneous tumors defined by characteristic molecular alterations. For example, *CIC::DUX4* and *EWSR1::PATZ1* sarcomas typically arise in deep soft tissue and rarely in bone (12, 18, 34, 35). Conversely, *EWSR1::NFATC2*, *FUS::NFATC2*, and *BCOR::CCNB3* sarcomas are primarily osseous (14, 19, 34, 36), while *BCOR*-ITD sarcomas show a distinctive infantile soft-tissue predominance (20). These site-specific tendencies provide valuable diagnostic context but must be interpreted alongside histopathologic and molecular findings.

Extraskeletal ESs occurred in relatively older patients (mean 26.2 years), roughly a decade older than skeletal cases (17.6 years), mirroring previous reports (28, 37). In contrast, non-Ewing SRCSs spanned a wider age range, including two infantile cases initially suspected to represent extraskeletal ES but reclassified on molecular grounds. One was an intramuscular gluteal tumor in a 5-month-old girl with a *BCOR* exon 15 partial duplication (non-canonical alteration); she achieved complete remission after multimodal therapy with excellent histologic response and remains disease-free at 4.8 years. The second, a gluteal-perineal neonatal tumor harboring *YWHAE::NUTM2B*, followed an aggressive course with death at 15 months. *BCOR*-ITD and *YWHAE*-rearranged sarcomas define an infantile SRCS group with truncal/abdominopelvic predilection and aggressive behavior; they share morphology and immunophenotype (BCOR, cyclin D1, SATB2) with clear-cell sarcoma of the kidney (20, 38, 39). Of note, classical ES in infants <1 year remains exceedingly rare, comprising ~2% of pediatric ES in SEER data (40).

Visceral ESs are uncommon but have been described in nearly all organ systems (41–45). In this cohort, visceral ES accounted for 10.5% of cases (8/76), involving kidney (n=5), small intestine, anorectum, and retroperitoneum. These tumors predominantly affected adults (mean 34.8 years) and often required extensive immunohistochemical and molecular work-up. Additional cases in breast and oral mucosa, though not strictly visceral, posed similar diagnostic challenges due to their atypical presentation. Most tumors harbored *EWSR1::FLI1*, aligning with canonical Ewing-family genetics. Renal ES was the most frequent visceral site, consistent with reports showing large tumor size, infiltrative growth, and aggressive clinical behavior (46–49), often with intravascular extension into the renal vein or IVC (50, 51). One renal case progressed rapidly with brain metastases.

Adult-onset extraskeletal ES often presents significant diagnostic pitfalls due to the broad differential diagnosis, especially when accompanied by non-classic morphologic and immunophenotypic features. Variant architectures (nested, epithelioid, rhabdoid) may prompt misclassification as carcinoma, melanoma, lymphoma, or another sarcoma (37, 52). Aberrant cytokeratin (53), synaptophysin, or S100 expression are also recognized ES pitfalls and can be especially misleading in extraskeletal or visceral settings. Here, some renal ESs showed pseudopapillary/perivascular patterns with patchy cytokeratin expression mimicking renal cell carcinoma; a breast ES resembled primary solid papillary carcinoma; and a rectal ES with extensive cytoplasmic clearing simulated metastatic clear cell carcinoma. Additionally, two extraskeletal tumors were excluded after review confirmed metastatic melanoma and metastatic carcinoma of unknown origin. These examples reinforce the need for robust molecular testing in adult undifferentiated round-cell tumors, especially at atypical anatomic sites.

Stage at diagnosis was the dominant survival determinant regardless of location, with metastatic presentation predicting inferior outcomes in line with prior studies (54, 55). Skeletal and extraskeletal ESs showed comparable survival, echoing data that site does not significantly affect outcome when standardized multimodal therapy and adequate local control are achieved (56–58). Long-term survival following relapse remains poor; however, in this cohort long-term estimates are limited by recent case accrual and substantial censoring (59). Additional prognostic factors include age, tumor volume, and histologic response to chemotherapy (60, 61). Molecular correlates of prognosis have also been increasingly described, including *STAG2* and *TP53* mutations, *CDKN2A* deletions, genomic copy-number alterations, and epigenomic signatures (2), and prior studies have shown that certain molecular subtypes within the non-Ewing SRCS spectrum may exhibit distinct clinical behavior and prognosis (8, 18). Despite advances in molecular classification, however, therapeutic strategies for undifferentiated SRCSs remain largely protocol-driven in current clinical practice, with most patients—including those with non-Ewing molecular subtypes—managed using ES-based multimodal regimens (8). While emerging approaches targeting *EWSR1::FLI1*-associated pathways and immunotherapeutic strategies are under active investigation, these remain largely investigational, and genotype-specific treatment paradigms have not yet been incorporated into routine clinical care (62–64).

As with most rare-tumor studies, several considerations should be noted. As a single-institution experience, sample size is inherently constrained—particularly given the overall rarity of sarcomas and the even lower incidence of non-Ewing SRCS subsets. In this context, a cohort of 90 molecularly characterized SRCSs accumulated over a decade represents a substantial dataset for a rare-disease population, although certain molecular subgroups remain small. Moreover, the non-Ewing cohort is heterogeneous, combining genetically defined tumors (e.g., *CIC::DUX4*, *BCOR-altered*) with fusion-negative undifferentiated SRCSs; this grouping was unavoidable due to limited case numbers, although these represent biologically distinct entities. Larger multi-institutional or registry-based cohorts incorporating comprehensive molecular profiling will be essential to improve statistical power across these emerging molecular subsets.

## 5 Conclusion

In summary, this study highlights the morphologic overlap yet molecular divergence between ES and the heterogeneous group of non-Ewing undifferentiated SRCSs. Although skeletal and extraskeletal presentations differed notably—particularly in patient age—these site-based distinctions did not translate into significant differences in clinical outcomes. Accurate subclassification of SRCSs requires integrated assessment of anatomic context, morphology, immunophenotype, and molecular findings. This combined approach reduces diagnostic ambiguity, guides appropriate management, and aligns nomenclature with evolving biological insights. Future multicenter cohorts from our region incorporating more comprehensive molecular profiling will be essential to refine prognostic stratification within these genetically defined subsets and to support precision-based management strategies.

## Data Availability

The data presented in the study are deposited in the Figshare repository at: https://figshare.com/s/2248cdbbf6a724f8cf1c, under the DOI: 10.6084/m9.figshare.30686198.
